# Obstructive sleep apnea treated with custom-made bibloc and monobloc oral appliances: a retrospective comparative study

**DOI:** 10.1007/s11325-016-1377-1

**Published:** 2016-07-05

**Authors:** Göran Isacsson, Clara Fodor, Magnus Sturebrand

**Affiliations:** Department of Orofacial Pain and Jaw Function, Västmanland County Hospital, Entrance 27, SE-721 89 Västerås, Sweden

**Keywords:** Obstructive sleep apnea, Mandibular advancement device, Cost of treatment, Treatment outcome

## Abstract

**Purpose:**

The primary purpose of this hypothesis-generating retrospective study was to compare the effect of monobloc and bibloc (Narval™) appliances on the apnea–hypopnea index (AHI) and the total cost of treatment during the first year of treatment.

**Methods:**

Obstructive sleep apnea (OSA) subjects treated with a monobloc or bibloc during two different time periods were identified from medical records and data were extracted. Subjects treated with either of the appliances passed the same primary examination, follow-up visits, and follow-up polygraphic examination. A 1-year clinical follow-up was made on the bibloc group.

**Results:**

The study analysis included 110 monobloc- and 55 bibloc-treated subjects with baseline mean AHI of 23 and 22, respectively. AHI responders (AHI < 10 and/or a ≥50 % reduction of baseline AHI) were seen at follow-up in 61 % of the monobloc group and 56 % of the bibloc group. The improvement of the AHI value was similar in the two groups, with mean declines of 12.7 and 13.8, respectively. The ODI (oxygen desaturation index), lowest SpO_2_, longest apnea, and the mean Epworth sleepiness scale (ESS) score were significantly reduced by 3.1 (monobloc) and 2.2 (bibloc), i.e., at the same level for both groups. The total direct cost of treatment for a 1-year treatment was 17 % higher for the bibloc-treated subjects than for the monobloc-treated subjects.

**Conclusions:**

The results indicate that the monobloc and bibloc appliances are equally effective but the cost of treatment over 1 year was higher with the bibloc. However, prospective randomized controlled trials are needed to adequately test the assumption that the two treatment modalities are equally effective.

## Introduction

The oral appliance (OA) is constructed so as to protrude the mandible during sleep and is a well-established treatment method of obstructive sleep apnea (OSA). The Food and Drug Administration (FDA) classifies intraoral appliances for snoring and/or OSA as class II (special controls) [1]. Oral appliances are indicated for use in patients with mild to moderate OSA who prefer them to continuous positive airway pressure (CPAP) therapy, or who do not respond to, are not appropriate candidates for, or who fail treatment attempts with CPAP [2]. A number of different designs of the OA are fabricated today, but there are two main types, namely the monobloc and the bibloc appliance. The former is a solid block of acrylic retained with clasps on the teeth and keeps the jaws in a fixed closed protruded position. The bibloc has separate constructions for the upper and lower jaws, equipped with connectors that protrude the mandible. The bibloc appliance allows the dentist to adjust the mandibular protrusion chairside, while the monobloc appliance requires the support of a dental technician. It is therefore assumed that the bibloc appliance should take less time to fit.

In a systematic review, Ahrens et al. [3] presented two studies comparing one-piece with two-piece appliances, in which no difference in reduction of AHI. These results are in contrast to the findings of a retrospective study by Lettieri et al. [4], who found significant advantages in AHI reduction with adjustable compared with fixed appliances. In a systematic review, Serra-Torres et al. [5] also concluded that adjustable and custom-made mandibular advancement appliances give better results than fixed and prefabricated appliances, and that monobloc appliances give rise to more adverse events.

At the beginning of 2011, the Narval™ bibloc appliance (hereafter, the bibloc appliance) was introduced to the Swedish market. The compliance, efficiency, side effects, and true cost of treatment for the bibloc in comparison with the commonly used monobloc appliance were not known. The primary purpose of this hypothesis-generating exploratory retrospective study was to compare the effect of monobloc and bibloc appliances on the apnea–hypopnea index (AHI), and the cost of treatment during the first year of treatment. A clinical- and questionnaire-based evaluation was also made on the bibloc-treated group after 1 year.

## Materials and methods

### Subjects

Subjects had an established diagnosis of OSA and had been referred to the Department of Orofacial Pain, Vastmanland Hospital, Västerås, for treatment with an oral appliance. All subjects, independent of subjective symptoms, who commenced treatment with monobloc appliances during the period January 2009 to May 2010, and all subjects who commenced treatment with bibloc appliances during the period January 2011 to January 2012 were included. The monobloc appliances were all fabricated by Boxholm Tandteknik^1^ and the bibloc appliances were manufactured by ResMed.^2^

Inclusion criteria were a diagnosis of OSA with AHI ≥ 10, and an oral status allowing retention of an appliance. Exclusion criteria were snoring without OSA, maximal protrusion of less than 5 mm, and ongoing prosthodontic treatment.

The treatment in all phases including all technician costs for all subjects was covered by the national health security program (the annual cost for an adult person is at a maximum of 1100 skr for all health services).

### Study procedure

This is a clinical retrospective open effectiveness study describing polygraphic respiratory data and the cost of treatment for the year after the commencement of treatment with monobloc and bibloc appliances. All subjects treated with a monobloc appliance were contacted by mail to obtain their written informed consent to extract information from their medical records. No further clinical study examination of the monobloc group was made. Information was collected from the subjects’ baseline data and during the year after commencing the treatment. Data from the bibloc group were gathered in the same way but the subjects were also invited to participate in a 1-year follow-up. The clinical management of all subjects, independent of type of appliance, was the same at primary examination, follow-up, and polygraphic examination at baseline and follow-up.

All subjects visited the clinic, where a baseline clinical examination was made and impressions of the jaws were taken. A protrusion index protruded the mandible to 60–80 % of the maximal protrusion. Treatment commenced 2–3 weeks after the baseline examination and the appliance was fitted into the mouth. If the retention was insufficient or needed adjustment, it was returned to the technician for adjustment or redesign.

An evaluation visit was scheduled 1–2 months after the treatment commenced. Without any specified protocol, the subject was asked about snoring/apnea and the effect of the appliance and ESS score collected. If required, the appliance was adjusted. To adjust the degree of protrusion, the monobloc was returned to the technician together with a new protrusion index and an extra visit was then scheduled. Protrusion adjustment of the bibloc appliance was made chairside. If the subject experienced pain, discomfort, or problems with adapting to the appliance, individual considerations were made.

Polygraphy was performed with concomitant use of the appliance after the clinical evaluation. In cases of unsatisfactory respiratory values (AHI > 10), the mandible was advanced if possible. Those who then had the appliance adjusted were subjected to additional polygraphy.

The bibloc appliance treatment subjects were invited to participate in a clinical follow-up examination and to respond to a questionnaire 1 year after treatment commenced.

### Somnopolygraphic examination

Polygraphy was completed at the Department of Physiology, Vastmanland Hospital, Vasteras, as part of the clinical routine for the examination of sleep apnea. All subjects underwent a one-night respiratory baseline polygraphy (Embletta®, ResMed) without any respiratory support. After 5–6 months of use of the appliance, the polygraphy was repeated with the appliance in place.

### Cost of treatment

The calculation of a 1-year direct total cost of treatment for the payer (the County Council) was based on the appliance fabrication cost (technician cost) plus the dental office time, which was standardized to 1 h for the first visit, 30 min for the second visit (treatment start), and 30 min for the third visit (follow-up). All extra visits, including also those for dental laboratory adjustment of protrusion, were set at 30 min each. The cost of the dental office time was fixed at 2000 Swedish krona (SEK) (€216, $235) per hour.

### One-year follow-up clinical examination and questionnaire

A clinical examination was made at the 1-year follow-up of the bibloc appliance. The patient was asked to bite with a mild force. Occlusal stability in the intercuspal position was registered if the molar teeth could keep a firm grip on an occlusal foil; if not, it was classified as posterior open bite. Masseter and temporalis muscle palpation tenderness and pain with jaw movements were recorded with a yes/no response. The TMJ condylar movement, tenderness to palpation, and sound- and jaw movement-related pain were also registered as yes/no. The maximal mouth opening (distance between the incisors) and the maximal range of protrusion in the premolar region were measured.

The subjects completed a questionnaire with items on jaws, teeth, snoring, and sleepiness. In addition, sleepiness was also graded on an 11-point scale where (0 = no sleepiness; 10 = worst imaginable sleepiness). The question “How important is the appliance for your and/or your bed partner’s well-being” was measured using an 11-point Likert scale (0 = no importance; 10 = greatest possible importance). Patients rated the change in their overall status since the beginning of the study treatment on the seven-point Patient Global Impression of Change (PGIC) scale (1 = very much improved; 2 = much improved; 3 = minimally improved; 4 = no change; 5 = minimally worse; 6 = much worse; 7 = very much worse).

### Statistics

Because of the exploratory and retrospective nature of the study, no power calculation was made. Instead, reasonable sized groups of subjects were included to allow proper hypothesis generation. The results were presented as descriptive statistics and tabulations. The primary variable was the difference between the baseline and follow-up polygraphic AHI value. Secondary variables were the cost of 1 year of treatment, adverse events, and other polygraphic measures.

The Uppsala Regional Ethical Review Board, Sweden approved the study 12th September 2012, #2012/307. Informed consent was obtained from all subjects in both the mono- as well as the bibloc groups.

## Results

**Fig. 1:**
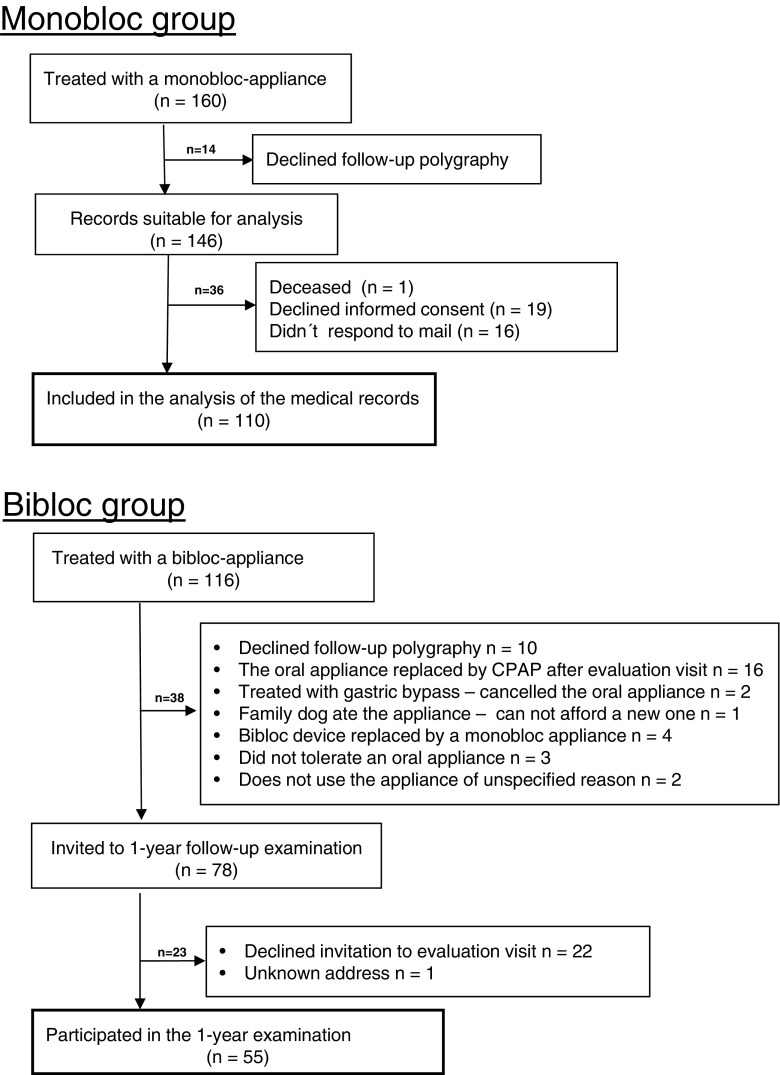
Recruitment schedule of the subjects treated with monobloc and bibloc appliances

During the defined period, 160 subjects had a monobloc appliance fitted. All but one subject (deceased) were sent a letter asking for informed consent to extract data from their medical records. Up to two follow-up letters were sent to those who did not initially respond. Figure 1 illustrates the recruitment flow for the 110 subjects who were included in the study analysis. Ninety percent of the subjects (*n* = 99) received an appliance made of hard acrylic and the remaining 10 % received a soft elastomer appliance. During the defined period, 116 subjects had a bibloc appliance fitted (see Fig. 1). A total of 55 subjects were included in the study and visited the clinic for the 1-year follow-up.

The baseline demographics, mandibular movement range, and polygraphic data are described in Table 1. The proportion of males was 79 % in the monobloc group and 80 % in the bibloc group. The age range was 31–82 years (mean = 58) for the monobloc group, and 37–76 years (mean = 57) for the bibloc group. The two treatment groups were similar in most baseline parameters except for the maximal protrusion range, and consequently the degree of mandibular advancement with the appliance. The bibloc appliance group had the mandible protruded 3 mm on average more than the monobloc group. CPAP treatment failure preceded 15 % of treatment in the monobloc group and 8 % in the bibloc appliance group. One subject in each group had been subjected to oropharyngeal surgery. The mean time from starting the bibloc treatment to the 1-year clinical follow-up ranged from 359 to 643 days with a mean of 481 days (16 months).

**Table 1:** Baseline characteristics of subjects treated with a sleep apnea oral appliance

	Monobloc appliance *n* = 110 mean (SD)	Bibloc appliance *n* = 55 mean (SD)
Subject characteristics		
BMI	28 (4.6)	28 (4.2)
ESS	11 (5.1)	10 (4.2)
Characteristics of somnopolygraphy		
ODI	21 (11.1)	20 (13.0)
AHI	23 (11.2)	22 (14.8)
Lowest SpO_2_ (%)	81 (6.4)	82 (6.2)
Longest apnea (sec)	46 (20.0)	42 (23.2)
Characteristics of mandibular protrusion		
Maximal mandibular PTR range (mm)	10 (2.6)	12 (2.8)
Mandibular PTR with appliance (mm)	6 (2.0)	9 (2.1)
Proportion of appliance-guided PTR in relation to max PTR (%)	67 (9.0)	73 (5.6)

*BMI* body mass index, *ESS* Epworth sleepiness scale, *ODI* oxygen desaturation index, *AHI* apnea hypopnea index, *SpO*_*2*_ oxygen saturation, *PTR* protrusion

At the beginning of treatment, 95 % of both the monobloc and bibloc appliances could be fitted into the mouth and jaws; the remaining 5 % of appliances required technician support for adjustment or had to be remade.

At the evaluation visit, 11 subjects (10 %) in the monobloc group needed to have their appliance adjusted to better control the effect on apnea and/or snoring. These subjects were subjected to a new protrusion index and the technician adjusted the appliance accordingly. In the bibloc group, 21 subjects (38 %) had the length of the connector rod exchanged, thereby correcting the degree of protrusion. This was done chairside, without the support of a technician.

At the evaluation visit, 78 % of the monobloc and 67 % of the bibloc appliance-treated subjects were symptom free from the jaws and teeth. The remaining subjects had various symptoms as shown in Table 2.

**Table 2:** Symptoms of the teeth/jaw at the evaluation visit

	Monobloc *n* = 106 *n* (%)	Bibloc *n* = 55 *n* (%)
No subjective symptoms	86 (78)	37 (67)
A lesser degree of symptoms, which did not reduce the use of the appliance	14 (13)	9 (16)
Moderate degree of symptoms, which reduced the use of the appliance	5 (5)	7 (13)
Severe symptoms, which did not allow the use of the appliance	1 (1)	2 (4)

The use of an oral appliance significantly improved the respiratory parameters in both appliance groups. The AHI score reduced by a mean of 12.7 and 13.8 for the monobloc and bibloc appliance groups, respectively. The daytime sleepiness score, measured by the ESS, was significantly reduced with the use of the appliances (Table 3).

**Table 3:** Improved difference from baseline without any appliance vs. with the use of an appliance

	Monobloc appliance			Bibloc appliance		
	Mean difference (SD)	95 % CI	*p* ^a^	Mean difference (SD)	95 % CI	*p* ^a^
AHI	12.7 (11.6)	10.5;14.8	0.000	13.8 (14.8)	9.8;17.8	0.000
AI	8.3 (9.1)	6.6;10.1	0.000	8.0 (1.6)	4.8;11.3	0.000
ODI	11.0 (11.3)	8.9;13.2	0.000	12.2 (13.7)	8.4;16.1	0.000
Lowest SpO_2_ (%)	3.0 (6.1)	1.7;4.2	0.000	2.1 (5.6)	0.5:3.6	0.000
Longest apnea (sec)	15.2 (19.4)	11.1;19.3	0.000	11.0 (20.0)	5.0;17.0)	0.001
ESS	3.1 (4.0)	2.2;3.9	0.000	2.6 (4.3)	1.0:4.1	0.002

*AHI* apnea hypopnea index, *AI* apnea index, *ODI* oxygen desaturation index, *SpO*_*2*_ oxygen saturation, *ESS* Epworth sleepiness scale

^a^ *p* value reflects the difference within the treatment group

**Fig. 2:**
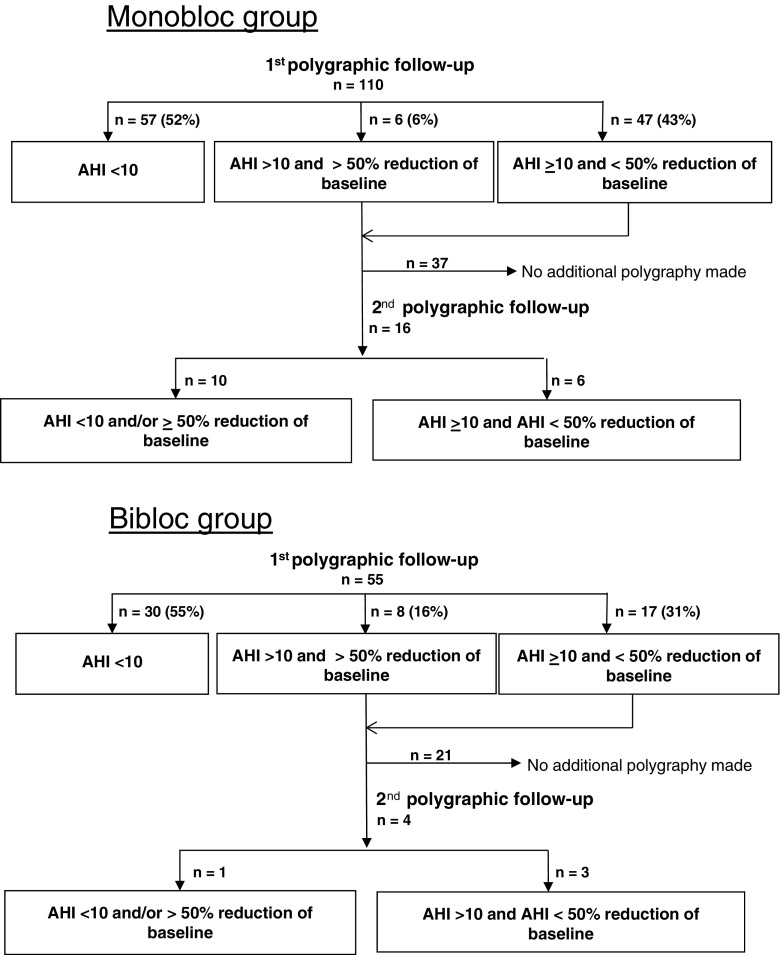
Polygraphic outcome following treatment with monobloc and bibloc appliances

Based on polygraphic data at follow-up, 57 subjects (52 %) in the monobloc group got an AHI < 10. Those with AHI ≥ 10 were recalled to the clinic and the mandibular protrusion was advanced if possible. As a consequence, 16 subjects had a second polygraphy, with an end result of 67 (61 %) AHI responders (AHI < 10 and/or 50 % reduction of baseline AHI) registered (Fig. 2).

Polygraphic examination of the bibloc group revealed 30 subjects (55 %) with an AHI < 10 with four subjects referred for a second polygraphic examination. A total of 31 (56 %) were classified as AHI responders.

During the year after starting the appliance treatment, the number of extra visits to the clinic was 0–6 occasions for both groups (mean = 1.17 for the monobloc group, 1.0 for the bibloc group). The total direct cost of treatment for the payer over 1 year could then be calculated as SEK 7270 (€786, $854) for a monobloc-treated subject and SEK 8500 (€920, $999) (∼17 % higher) for a bibloc-treated subject (Table 4) To note, the fabrication cost was fixed independent from number of adjustments to be done by the technician.

**Table 4:** The mean total treatment direct cost in Swedish crowns, skr, during one 1-year considering both the technician and dentist costs

	Fabricating cost skr	Cost for three routine visits at the dental office^a^	Mean number of extra visits during a year	Cost for extra visits to the dental clinic	Total cost during a year
Monoblock	2100	4000	1.17	1.17*0.5*2000 = 1170 skr	7270 skr
Biblock	3500	4000	1.0	1.0*0.5*2000 = 1000 skr	8500 skr

^a^Cost of dental office per hour was set to 2000 skr. Calculated dentist time—visit 1: 1 h for information, clinical examination, protrusion index, impression; visit 2: 0.5 h for fitting in the appliance; visit 3: 0.5 h for the follow-up, which sum up to 2 h. All extra visits calculated to 30 min

At the 1-year follow-up, the predefined questions revealed that one third of the subjects had stiffness/tiredness of the jaws and one fifth had orofacial pain and difficulties fitting the teeth after waking up (Table 5). The mean score in response to the question “How important is the appliance for you/your bed partner’s well-being” was 8.7 (SD 2.3) on the 11-point Likert scale (0 = no importance; 10 = greatest possible importance). The overall status at the 1-year follow-up since the beginning of treatment was rated at least “much improved” by 89 % of the subjects and none of the subjects scored worse on the PGIC scale. At the 1-year clinical examination, the most common clinical sign was TMJ sound and masseter and temporalis muscle palpation tenderness. A posterior open bite occurred in four of the subjects (Table 6).

**Table 5:** Positive response to a series of predefined questions on subjective symptoms at 1-year follow-up by the 55 subjects treated with a bibloc appliance

	*n* (%)
Stiffness/tiredness of the jaws	19 (35)
Difficulty in opening the mouth or chewing	6 (11)
TMJ sounds at chewing	10 (18)
Orofacial pain	11 (20)
Difficulty fitting the teeth after waking up	12 (22)
Difficulty fitting the teeth in the daytime	2 (4)
Disturbed by my own snoring	4 (7)
My snoring disturbs others	18 (33)
Disturbed/woken up by my own apnea	2 (4)
Bed partner worried about my apnea	3 (6)
Usually wake up during the night	35 (64)

**Table 6:** The outcome of the clinical examination at the 1-year follow-up of bibloc-appliance users, *n* = 55

	One side *n* (%)	Bilateral *n* (%)
Posterior open bite	3 (6)	1 (2)
Masseter and temporalis muscle palpation tenderness	8 (15)	8 (15)
TMJ palpation tenderness	1 (2)	4 (7)
Impaired translation of condyle	2 (4)	2 (4)
TMJ sound	9 (16)	5 (9)
TMJ pain at jaw movement	2 (4)	2 (4)
Jaw muscle pain at jaw movement	1 (2)	2 (4)

## Discussion

The results of the present retrospective hypothesis-generating study indicate that the monobloc and bibloc appliances show the same level of efficiency in reducing apnea–hypopnea events, sleepiness, and the degree of side effects. There was one major difference, namely the cost of treatment, with the bibloc appliance being ∼17 % more expensive than the monobloc appliance over 1 year. However, the results should be treated with caution for a number of reasons: the retrospective study design, the subjects were not recruited identically, the withdrawal rate was substantial, the study was not blinded and polygraphy was done and not polysomnograhpy, the adherence was not tracked, and the subjects were not randomly allocated to a treatment group.

Both the monobloc and bibloc appliances significantly reduced the AHI by a mean of about 12–14 events per hour, which is the same magnitude previously reported in both individual studies and systematic reviews [3, 6–8].

In a Cochrane meta-analysis, Lim et al. [9] analyzed crossover studies comparing active oral appliances with control appliances and found a reduction of AHI value by a mean of 15.15 events per hour. In parallel group studies, a mean reduction of 10.78 was noted. In studies comparing oral appliances with no treatment, the AHI improvement was a mean of 11 events per hour (95 % CI, 8–15) [10]. Vecchierini et al. [11] used Narval bibloc appliances in 369 OSA patients and found AHI reduction means of 7, 15, and 25 for mild, moderate, and severe OSA, respectively. This highlights the importance of presenting efficacy data based on OSA severity.

In a systematic review, Ahrens et al. [3] presented two studies [12, 13] comparing one-piece with two-piece appliances, in which no difference in reduction of AHI was found. Rose et al. [13] compared the Silencer® Herbst-type appliance with a Karwetzky activator on patients with mild OSA in a crossover study. Both appliances reduced the RDI value by 9 and 11 events per hour, respectively. In a crossover study, Bloch et al. [12] evaluated a monobloc and a Herbst appliance in moderate OSA patients. Although the reduction of AHI was similar, with 14–15 events per hour, the patients preferred the monobloc to the Herbst construction. These results are in contrast to the findings of a retrospective study by Lettieri et al. [4], who found significant advantages in AHI reduction with adjustable compared with fixed appliances. They also presented the results grouped by grade of severity and noted that in mild OSA, the effects of the two types of appliances were similar. In a systematic review, Serra-Torres et al. [5] concluded that adjustable and custom-made mandibular advancement appliances give better results than fixed and prefabricated appliances, and that monobloc appliances give rise to more adverse events. Objective polygraphic registrations are crucial as described by Marklund et al. [14] who evaluated fixed OAs incrementally advanced in response to the clinical observation. Whether or not adjustable oral appliances improve the effect more than monobloc appliances remains uncertain and prospective randomized controlled trial evaluations are required.

In the present study, 61 % percent of the monobloc group and 56 % of the bibloc group were classified as responders. This is less than the 75 % for monobloc and 67 % responders reported by Bloch et al. [12] for the Herbst bibloc appliance. The reason for the diverging results depends on a number of factors, including the severity of OSA, the degree of mandibular advancement, and the degree of vertical increase, as well as insufficient statistical power of the studies. Treatment effects tend to be greater in crossover trials than in parallel group studies, and the effects in trials of short duration are greater than in those in longer studies [15].

The ESS scores improved significantly in our study, with a mean reduction of 3.1 and 2.6 for the monobloc and bibloc groups, respectively. In a meta-analysis of randomized controlled parallel group studies, Lim et al. [9] compared active and controlled oral appliances showing an improvement of the ESS score by –2.09. Qaseem et al. [10] found that oral appliances improved the ESS score by 1.2 and 1.95 points, compared with no treatment and sham treatment, respectively. However, their meta-analysis did not include the more recent publication by Marklund et al. [16]. At a 4-month follow-up, they found that the ESS was significantly improved in both the active and the placebo appliance groups although the difference between the groups was not significant.

Martinez-Gomis et al. [17] followed patients using a Herbst-type bibloc appliance over 5 years and noticed that both symptoms and technical complications were more frequent during the first year of treatment, with a mean of 3.0 unscheduled visits. In our study, means of 1.17 and 1.0 extra visits were found for the monobloc and bibloc appliance groups, respectively. Although the difference in unscheduled visits may depend on differences in the strength of the constructions, it certainly depends on the variation in routine at the clinic and perhaps the variation in the primary advancement of the mandible at the start of the treatment. A potentially higher threshold for adjusting a monobloc appliance may also affect the number of visits. The timeframe for the cost analysis in the present study was only 1 year and of course the devices durability over a number of years may affect the cost in any direction. In Lim et al.’s Cochrane study [9], participants given the active oral appliance suffered side effects more frequently than those given the control appliance. In our study, one third responded that they had tiredness/stiffness of the jaws that frequently created difficulties in fitting their teeth after waking up. The most alarming objective finding of our study was the development of a posterior open bite in four of the 55 bibloc-treated patients after 1 year (the monobloc group did not go through a 1-year clinical follow-up). This highlights the need for a regular clinical check when mandibular advancement appliances are used.

The results of our study revealed a cost difference of SEK 1230 (GBP 95, $145, €132) over 1 year between the monobloc and bibloc appliances. For the payer, the GBP 95 difference may be of some importance as the two types of appliances are equally effective.

## Footnotes

1 Boxholm Tandteknik, Box 103, 590 10 Boxholm, Sweden.

2 ResMed Sweden AB, JanStenbecks Torg 17, 164 40 Kista, Sweden.
